# Developments in the Treatment of Midshaft Clavicle Fracture After Plate Fixation Failure: A Case Report with 2.5 Years of Follow-Up

**DOI:** 10.3390/reports9030250

**Published:** 2026-08-01

**Authors:** Sahar Ahmed Abdalbary, Sherif M. Amr, Ahmed Al-Feeshawy, Ehab A. A. El-Shaarawy, Khaled Abdelghany, Ahmed Abdel Moghny, Mohamed Abdel-Wahed

**Affiliations:** 1 Department of Orthopaedic Physical Therapy, Faculty of Physical Therapy, Nahda University, Beni Suef 11787, Egypt; 2 Department of Orthopaedic Surgery, Faculty of Medicine, Cairo University, Giza 12613, Egypt; 3 Department of Anatomy and Embryology, Faculty of Medicine, Cairo University, Giza 12613, Egypt; 4 Advanced Manufacturing Division, The Central Metallurgical Research and Development Institute, Cairo 11421, Egypt; 5 Mechanical Design and Production Department, Cairo University, Giza 12613, Egypt

**Keywords:** clavicle, midshaft fracture, 3D prosthesis, polyamide

## Abstract

**Background and Clinical Significance:** Clavicular fractures account for approximately 2.6% of all fractures, with 80% of clavicular fractures occurring in the middle-third of the bone. The middle-third of the clavicle lies directly under the skin without any protection from soft tissue or muscle attachments. The purposes of this case report are as follows: (1) to represent the prosthesis and its operative implantation and (2) to assess the radiological and clinical outcomes of using the prosthesis after a 2.5-year follow-up of the patient; **Case Presentation:** We present the case of a midshaft clavicle fracture in a 26-year-old, right-handed, male patient following a failure of conservative and open reduction and internal fixation of the fracture. A three-dimensional (3D) customized prosthesis with polyamide was designed for the patient. The patient presented 2.5 years after surgery with a VAS score of 2 and a DASH score of 21, and radiographic evaluation revealed good prosthesis position and no evidence of a stress fracture or unanticipated complications; **Conclusions:** This is a single case with successful polyamide prosthesis fixation in the treatment of a midshaft clavicle fracture after the failure of plate fixation.

## 1. Introduction and Clinical Significance

Clavicular fractures account for approximately 2.6% of all fractures, with 80% of clavicular fractures occurring in the middle-third of the bone [1]. The middle-third of the clavicle lies directly under the skin without any protection from soft tissue or muscle attachments. This thinnest area of the clavicle is a highly vulnerable site to trauma [2]. There is a high incidence of symptomatic malunion and nonunion after non-operative treatment of displaced midshaft clavicular fractures, and treatment options are still controversial [3].

In a study by [4] an all-operative approach was not deemed superior to an all-non-operative approach. Although the short-term functionality within 6 weeks seemed greater following operative management, this advantage was not seen at 1 year. Similarly, the risk of nonunion was significantly higher with non-operative treatment. However, all-operative procedures to decrease nonunion risk may lead to unnecessary operation and are not preferred [4].

Therefore, treatment choice is often made on a case-by-case basis considering specific fracture characteristics, patient comorbidities, functional level, and activity goals [5].

A number of biomaterials are used to construct bone scaffolds to rebuild the bones exposed to trauma. Polyamide, a synthetic polymer, offers good mechanical strength to the scaffold and exhibits excellent thermal and mechanical properties, along with biocompatibility. This makes it a strong candidate for tissue engineering [6].

Polyamides have been a biomaterial of choice for a long time, and they have been broadly developed for suture, membrane, and vascular applications due to their hardness, low cost, and outstanding biocompatibility. Polyamides are also extensively used in bone engineering due to the material’s high strength and flexibility, which results in high degrees of both crystallinity and hydrogen bonding [7].

Three-dimensional (3D) computed tomography (CT)-based software for operative planning has been extensively applied in the biomedical field to create a surface model of the prosthesis, simulating the healthy, contralateral side. Finite element analysis is helpful for testing the biomechanical analysis of the clavicular reconstruction plate. This method calculates the amount of force provided by the muscles as a consequence of the operative technique [8].

We report a case of midshaft clavicular fracture, which was successfully treated with a polyamide prosthesis to achieve stabilization for un-fused bone after failed conservative treatment and plate fixation. The purposes of this case report are as follows: (1) to represent the prosthesis and its operative implantation and (2) to assess the radiological and clinical outcomes of using the prosthesis after a 2.5-year follow-up of the patient. To our knowledge, a case report of this type has not been previously presented in the literature.

Written consent was obtained from the patient for the purposes of this publication.

## 2. Case Presentation

A 26-year-old, right-handed male presented to our hospital with a 17-day history of left-hand numbness, weakness, and pain in the left shoulder. The patient had a history of a midshaft clavicular fracture treated with plate fixation. The interval between the original fracture and plate fixation was 3 days. The interval between the initial plate fixation surgery and the subsequent reconstructive surgery with the customized polyamide implant was one and half year confirmed. Radiographic evaluation revealed a non-healed midshaft clavicular fracture. Preoperative pain assessment showed a visual analog scale (VAS) score of 7. The preoperative disability of the arm, shoulder, and hand (DASH) score was 85.3.

After the preoperative assessment, we removed the plates. A 3D CT scan showed the limit of the injury and the fracture status (Supplementary S1 Figure S1). A bulky callus around the fracture and a nonunion site was noted to be compressing the brachial plexus with a 23.6 mm gap and a 10.4 mm dislocation, which indicated plate fixation failure. Based on this, we decided to design a 3D customized polyamide prosthesis due to its durability and low cost.

### 2.1. Structuring of Prosthesis

3D customized prostheses have proven to be successful in a number of surgeries. A healthy clavicle 3D geometric design was created using data obtained from the 3D CT scan. The 3D customized design of the midshaft clavicular prosthesis was created using the normal anatomy from the healthy contralateral clavicle in 3D CT-based software for surgical planning, Mimics (Materialize NV, Leuven, Belgium). The prosthesis was fabricated using 3D selective laser sintering (SLS), which is a powder bed printing technology. Polyamide powder was used in the manufacturing of the prosthesis. The prosthesis consisted of a meshed plate with six pins placed on the two sides to close the prosthesis. The surfaces of the prosthesis were polished without any coating material. We created five models to find the one that most simulated the midshaft clavicular part of the clavicle to be fixed. Supplementary S1 Figure S1 shows the clavicular fracture from a coronal view and a transverse view. We refined the bone and removed the osteophytes marked by the red circle before the installation of the implant over the bone. Bone reorientation was carried out in the coronal and transverse views, and we built the implant over the bone. The implant had six fixation pins with a diameter of 2 mm. The implant had a mesh volume of 3 mm solid and 1 mm air. There was a clearance of 0.1 mm between the bone and the implant.

### 2.2. Finite Element Analysis (Supplementary Figures S2–S4)

After completion, the 3D-designed models were uploaded to the finite analysis software ANSYS Mechanical 2022 R2 (ANSYS Mechanical 2022 R2 Inc., Canonsburg, PA, USA) for analysis. Stress load allocation and distortion were computed by the software. Border line status was calculated at three fixed points—the terminals of the prosthesis on both sides and in the middle. The maximum stress was checked at the clavicular length that was most realistic with regard to clavicular fractures. After that, discrimination of the stresses expected and the load actually found by finite element analysis estimated that the outcome values were at a similar range in the simulation compared to real-world data. We established the result validity of FEA to investigate the outcome values extracted by the FEA ANSYS Mechanical 2022 R2 software. These calculations were carried out by comparing experimental data using another similar calculation technique.

Finite element analysis demonstrated that the maximum stresses within the fixation material remained below the material yield limit, indicating that neither plastic deformation nor structural failure would be expected under the simulated loading conditions.

### 2.3. Surgical Procedure

The implant was sterilized before surgery using an autoclave. Because dimensional precision was critical, the prosthesis was reassessed after autoclave sterilization. No detectable deformation or dimensional alteration was observed, confirming adequate thermal stability of the polyamide material.

**Figure 1:**
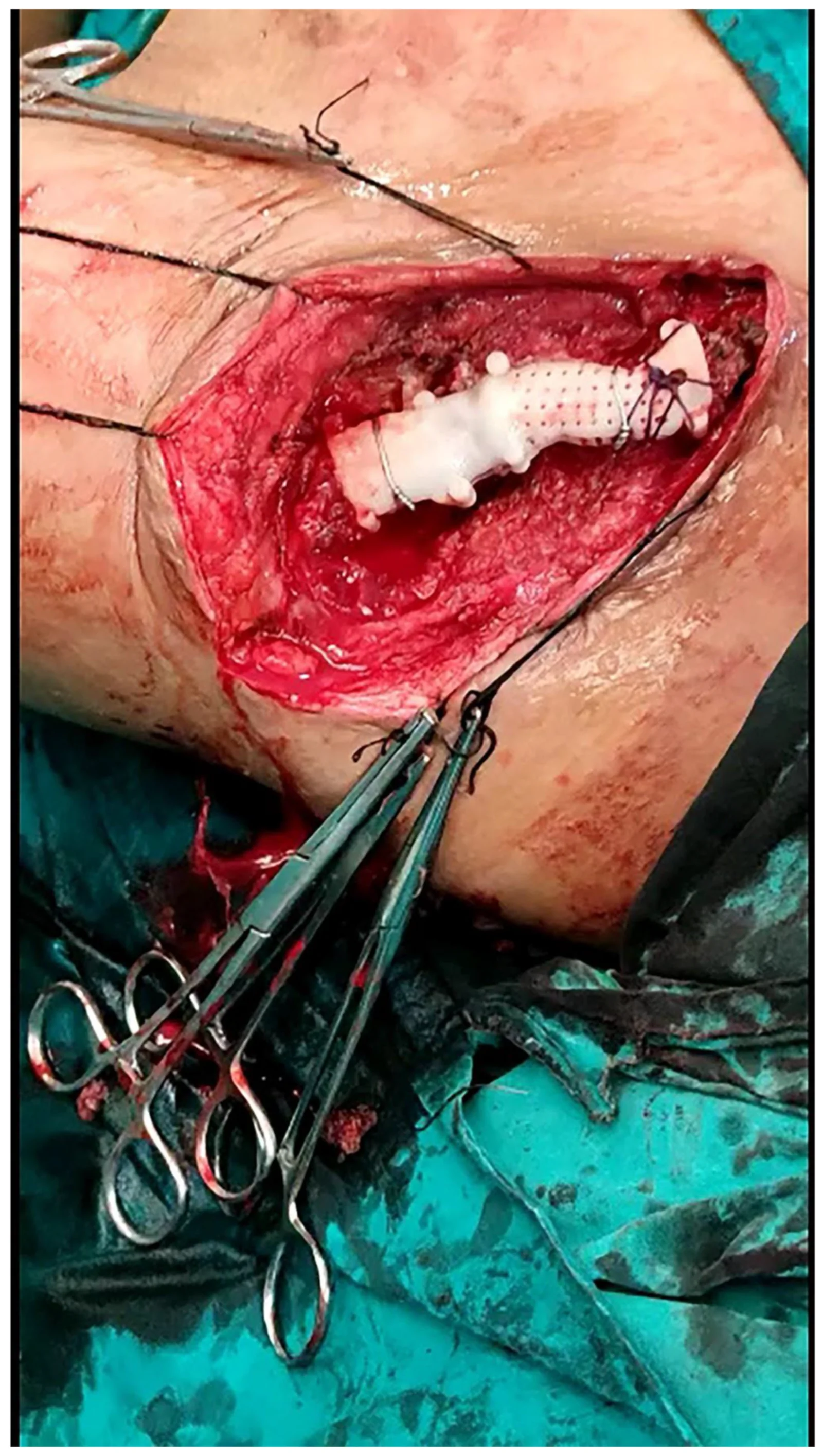
The Prosthesis while fixed at the clavicle.

The patient underwent surgery in a supine lying position. The operation site was sterilized, starting from the lateral border of the acromion to the sternum. Then, a horizontal skin incision was performed on the superior surface of the clavicle. The skin, platysma, and subcutaneous tissue were lifted with extreme care to protect the supraclavicular nerves from injuries. The fracture site was exhibited and viewed; then, callus resection and fracture reduction were performed due to the observed dislocation and gap (Supplementary S2 Video S1). The prosthesis was anatomically fitted to the residual clavicular segments and stabilized using six fixation pins integrated into the prosthesis design. Additional fixation was achieved using nonabsorbable sutures through designated holes in the implant for attachment to surrounding soft tissues (Figure 1).

There were no surgical complications.

### 2.4. Post-Operative Rehabilitation (Supplementary S1 Figure S5)

The shoulder was protected in a sling for 4 weeks. Codman’s pendular exercises and active assisted forward elevation and abduction were started immediately, but elevation and abduction were limited to 90 degrees for the first 4 weeks. After 4 weeks, restrictions were not placed on the range of movement. Strengthening and resistance exercises for the shoulder girdle were carried out from 6 weeks up to 12 weeks, supervised by a physiotherapist. The patient was already aware that his shoulder felt ‘more normal’ 3 weeks after the surgery.

Restriction of shoulder flexion and abduction to 90° during the first four postoperative weeks was intended to minimize mechanical loading across the clavicle–prosthesis construct during the early healing phase. Excessive shoulder elevation increases clavicular rotation and could generate larger bending moments and tensile forces across the implant–bone interface.

Therefore, restricting motion during the early postoperative period reduced the risk of excessive stress concentration, fixation failure, or implant micromotion, while allowing for the gradual adaptation of surrounding tissues.

### 2.5. Outcomes

At 3 weeks post-operation, the patient reported subjective improvement in shoulder comfort and daily functional use; however, formal functional assessment was not performed at that time.

Radiological and clinical assessments were performed at regular intervals post-operatively (Supplementary S1 Figure S6). At 2.5 years after surgery, the visual analog scale score was 2 under stress, and 1 at rest; the disability of the arm, shoulder, and hand score was 5.58; and radiographic evaluation revealed good prosthesis position and no proof of a stress fracture or unanticipated complications. Active range of motion (ROM) outcomes of the shoulder at 2.5 years after surgery showed 170° flexion, 170° abduction, 85° external rotation, and 65° internal rotation (Supplementary S1 Figure S5). When compared to the normal shoulder, muscle strength was 95% on flexion, 94% on maximum abduction, 94% on maximum external rotation, and 93% on maximum internal rotation. The patient was capable of returning to his job and the usual activities of his normal life and could participate in sports activities that need significant shoulder range of motion, like pulling and lifting weights. The patient was fully satisfied with his shoulder function.

## 3. Discussion

Our study introduced a case of a midshaft clavicular fracture that was successfully reconstructed using a 3D-printed polyamide prosthesis after initial management with failed plate fixation. Post-operatively, the patient was satisfied with his shoulder ROM, and no complications, such as infection or rejection, were noted. The procedure used for this case kept the supportive functions of the shoulder and protected the most important vessels and nerves under the clavicle.

There is a high prevalence of symptomatic malunion and nonunion after conservative treatment of displaced midshaft clavicular fractures, and treatment options are controversial [9].

Locking plate fixation, coracoclavicular fixation utilizing suture anchors or button devices, tension band wiring, transacromial pinning, and hook plate fixation are some of the surgical procedures that have been introduced to treat unstable distal clavicle fractures. Among these, hook plate fixation has been used extensively because it is very simple to apply in surgical procedures and can offer secure fixation, especially in instances with minor lateral fragments or concomitant coracoid fractures. Although this approach has been shown to have high union rates, using hook plates is sometimes linked to difficulties. These include peri-implant fractures, which frequently require implant removal following fracture healing; acromial erosion; severe postoperative stiffness; and subacromial impingement [10].

Polyamide is specifically engineered to allow for the fabrication of biologically inert implants using standard fused deposition manufacturing 3D printers. One example of the use of this material is in manufacturing custom implants for reconstructing the damaged cartilage of the knee [11].

Polyamide is widely utilized because of its remarkable mechanical, thermal, and electrical qualities. Polyamide is the best option for biomedical applications, such as dental implants, biomedical devices, scaffolds for bone formation, and orthopedic prosthesis, thanks to these qualities and its biocompatibility. Additionally, polyamide may be utilized in a variety of 3D-printing techniques, including selective laser sintering (SLS) and fused deposition modeling (FDM), which makes it the best option for orthoses with customized shapes [12].

Specialized implants can be designed specifically according to a patient’s body weight and structure, which are required iterations of models and tryouts. With the combination of 3D computer modeling and on-demand 3D printing, a patient can now leave the hospital with a pliable customized implant [13].

SLS is based on the fusion of powder. It employs a high-powered laser as a heat source to sinter powdered material (usually nylon, polyamide, or metals) to construct 3D parts layer by layer [14]. In contrast, FEM is a useful and efficient procedure to examine real-life conditions using real-time, expected borderline, and loading situations, specifically for biomedical purposes. Structural analysis is useful in this kind of research. It is not easy to analyze and predict the precise mechanical behavior of nearby tissues and implants in vivo during the repair stage in orthopedic applications. However, FEA is a critical and meaningful tool for structuring, examining and optimizing medical designs [1].

Polyamide offers several advantages, including lower weight, lower elastic modulus, flexibility closer to that of biological tissues, ease of customization through additive manufacturing, and lower manufacturing cost. In addition, polyamide demonstrates excellent biocompatibility and has been successfully utilized in various biomedical applications.

We acknowledge that titanium alloys possess superior fatigue strength, higher stiffness, and a longer history of clinical use [15]. Therefore, titanium remains the preferred material for load-bearing orthopedic reconstruction. However, in the present case, polyamide was selected because of its ability to produce a patient-specific implant with complex anatomical geometry while minimizing cost and manufacturing time [16]. Long-term mechanical fatigue testing is required before broad clinical application of this prosthesis.

Conservative management of a clavicular nonunion (failure of the bone to heal) typically relies on nonoperative strategies. When not considering a specific patient, it is best to examine the general nonoperative strategies and the specific clinical red flags that make surgery necessary [17].

This polyamide prosthesis was successful in the repair of the midshaft clavicular fracture, which had a bony loss after failure of plate fixation. The patient in this study achieved functional and appearance-related fulfillment due to this midshaft clavicular prosthesis. However, this type of procedure may not be suitable in a patient with contraindications to CT imaging or surgery.

Finite element analysis (FEA) is an effective computational method for evaluating different loading scenarios and design options. The findings of this study showed that the maximum stresses within the fixation materials stayed below their yield limits, suggesting that neither plastic deformation nor structural failure would occur under the applied conditions.

We acknowledge that dedicated porous structures optimized for osseointegration may further improve biological fixation and should be investigated in future studies.

This study concluded that using polyamide prostheses for the treatment of midshaft clavicular fractures is recommended if initial plate fixation has failed. As seen in our case, the advantage of the novel prosthesis was improved shoulder function with a return to daily living activities. Although this is a single case of a successful polyamide prosthesis fixation for a midshaft clavicular fracture with a bony defect, our outcome is promising and may benefit other similar cases.

Before large-scale clinical implementation, additional investigations are required, including static and cyclic fatigue testing, long-term wear and durability analysis, mechanical testing under physiological loading conditions, biocompatibility and tissue integration studies multi-center prospective clinical studies, and comparative studies with conventional reconstruction techniques and titanium implants.

## Data Availability

The data that support the findings of this study are available from the corresponding author upon reasonable request.

## Supplementary Materials

The following supporting information can be downloaded at https://www.mdpi.com/article/10.3390/reports9030250/s1. Supplementary S1 Figure S1: 3D CT Before operation and Manufacturing of the prosthesis; Supplementary S1 Figure S2: Explain Axial compression test; Supplementary S1 Figure S3: Cantilever bending test; Supplementary S1 Figure S4: Explain Torsion test; Supplementary S1 Figure S5: Active Range of motion of the shoulder joint after 2.5 years follow-up; Supplementary S1 Figure S6: Post operative X Ray. Supplementary S2 Video S1: Callus resection and fracture reduction.
