# Supplementary material for: Developments in the Treatment of Midshaft Clavicle Fracture After Plate Fixation Failure: A Case Report with 2.5 Years of Follow-Up
Source: Reports (MDPI). 2026 Aug 1;9(3):250. doi: 10.3390/reports9030250 (PMC13511425; doi:10.3390/reports9030250)
Supplement: Supplementary file 1 [file reports-09-00250-s001.zip › reports-4275925-supplementary.pdf]

Supplement S1 Figure S1: 3D CT Before operation and Manufacturing of the prosthesis

Segmented bone reconstruction:

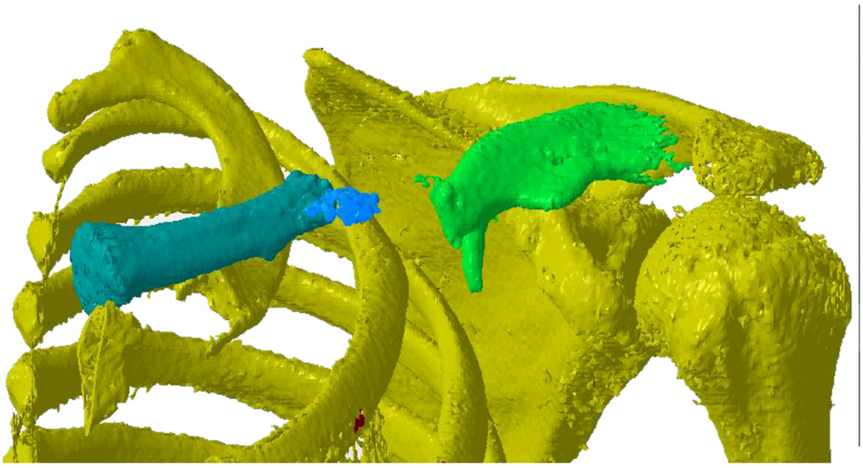

Coronal View

Segmented bone reconstruction:

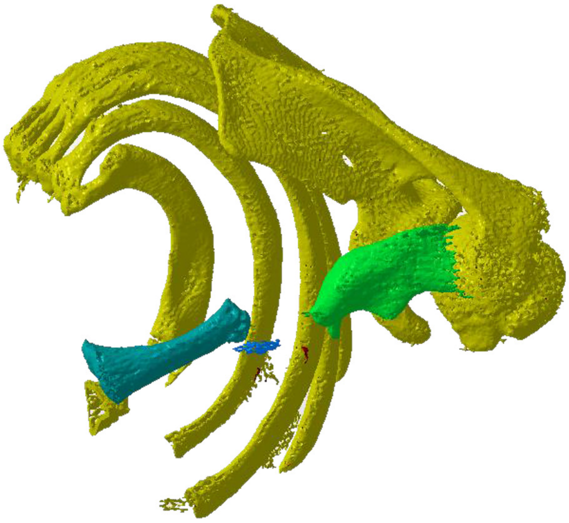

Transvers View

Segmented bone reconstruction:

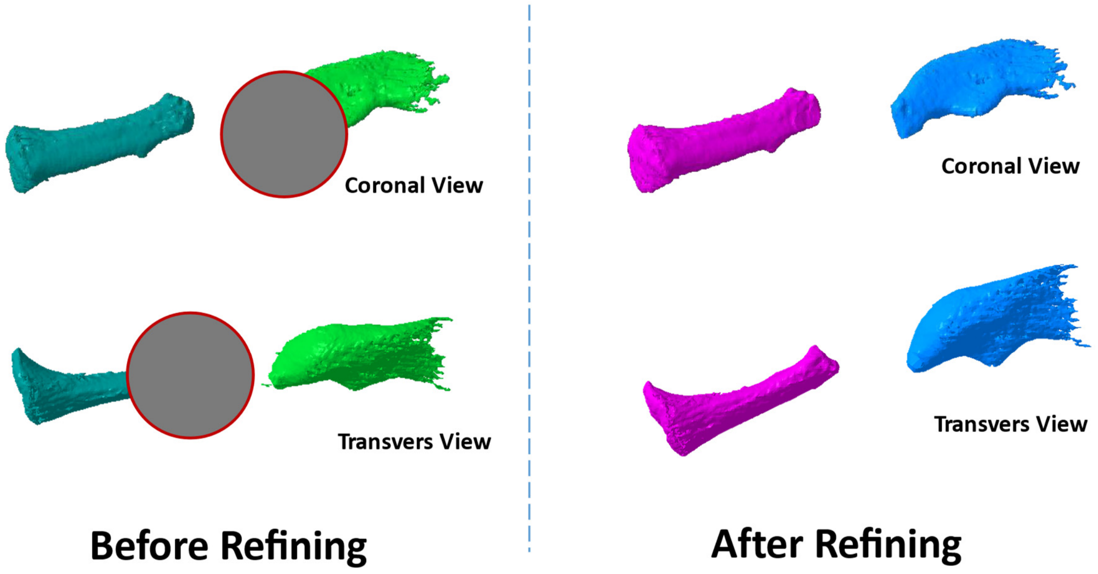

Segmented bone reconstruction:

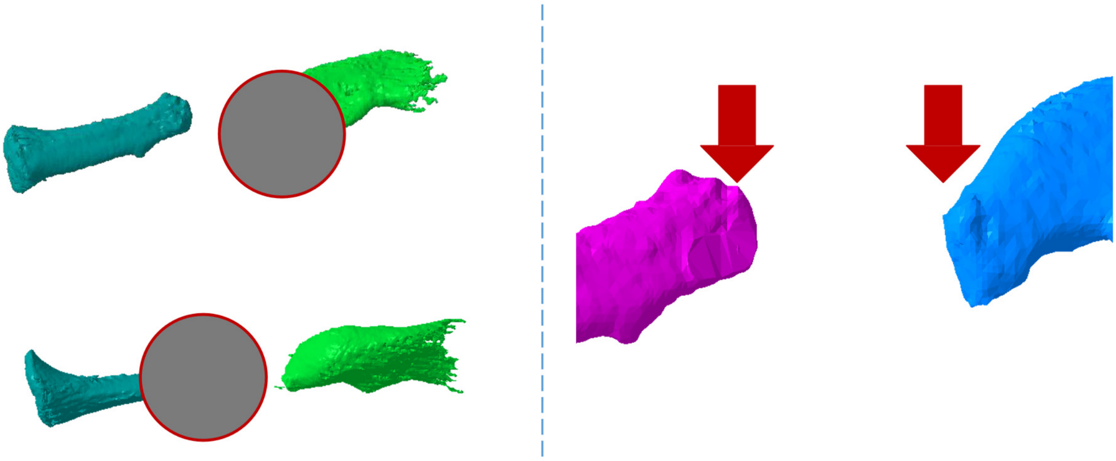

** Remove the osteophytes marked by the red circle before the installation of implant over the bone. The bone surface should be like shown in the right side figure

Segmented bone reconstruction: fracture gap distance

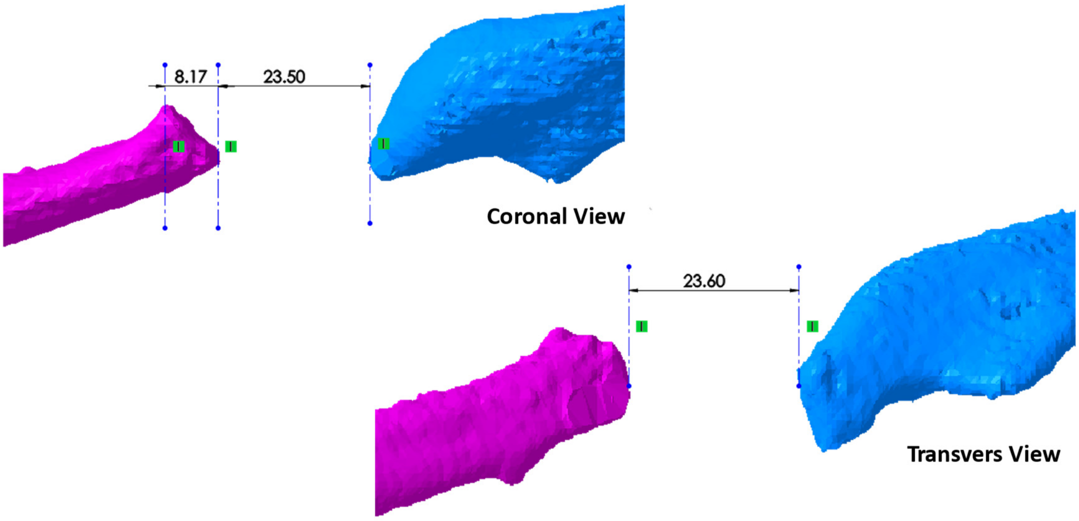

Bone reorientation:

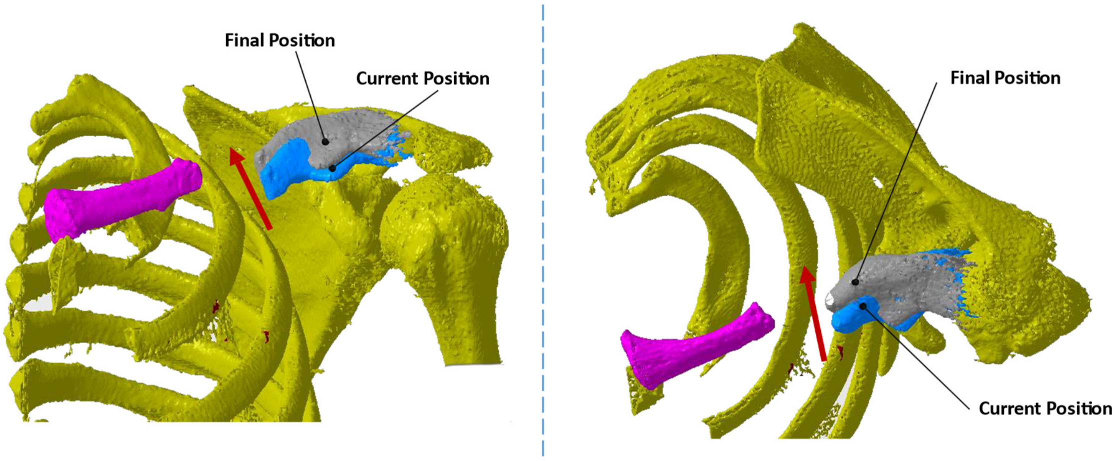

Coronal View | **Transvers View**

Bone reorientation:

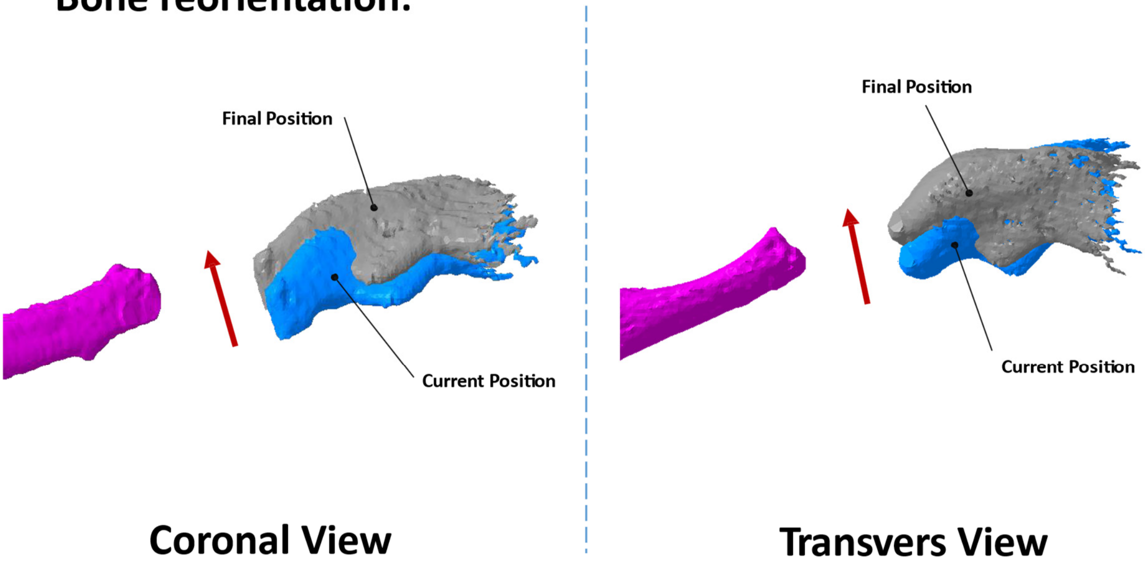

Coronal View

Transvers View

Implant over the bone

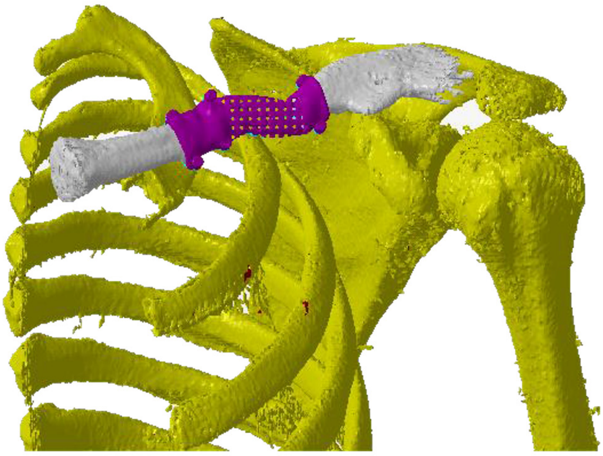

Coronal View

Implant over the bone

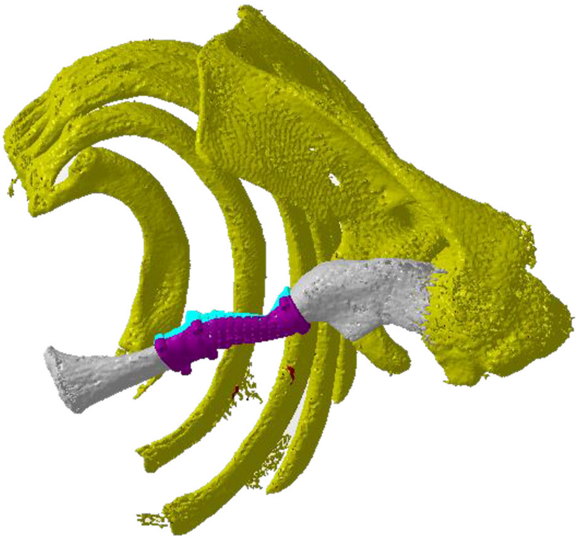

Transvers View

Implant over the bone

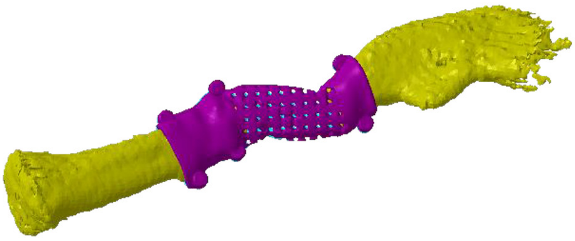

Coronal View

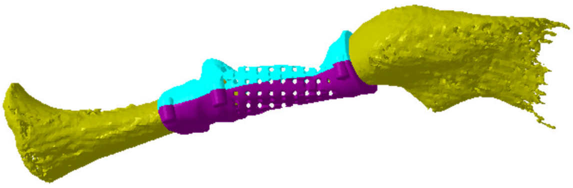

Transvers View

Implant over the bone

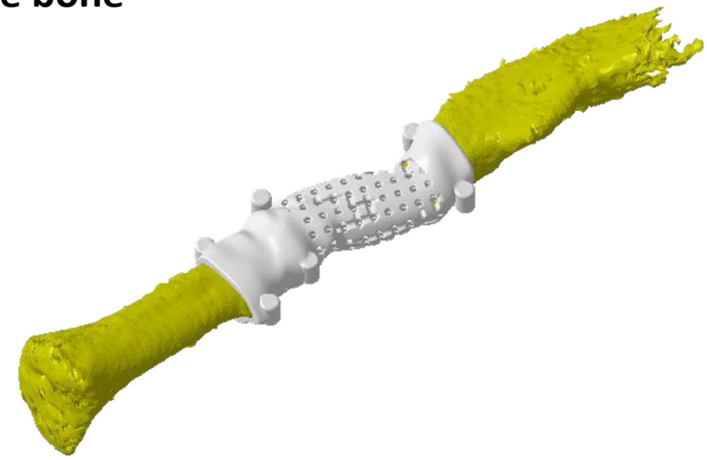

Isometric View

The Implant

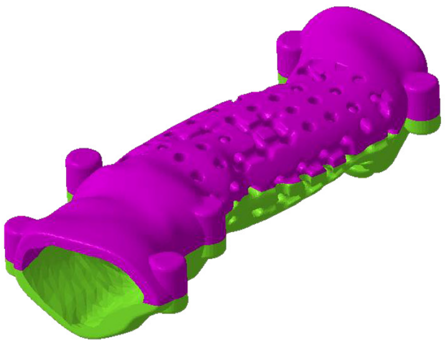

Medial Side

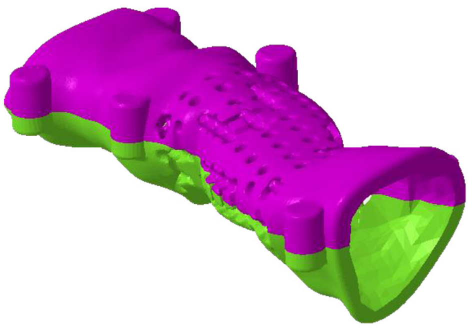

Lateral Side

The Implant: Lower Component

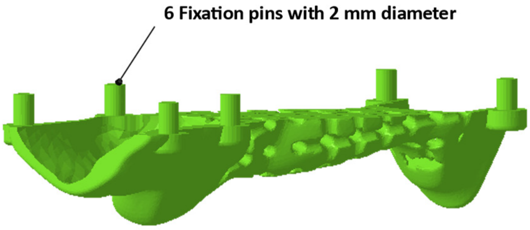

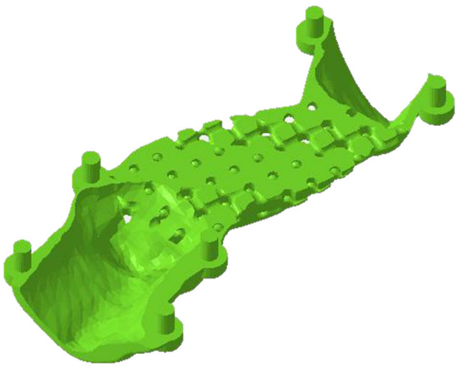

The Implant: Upper Component

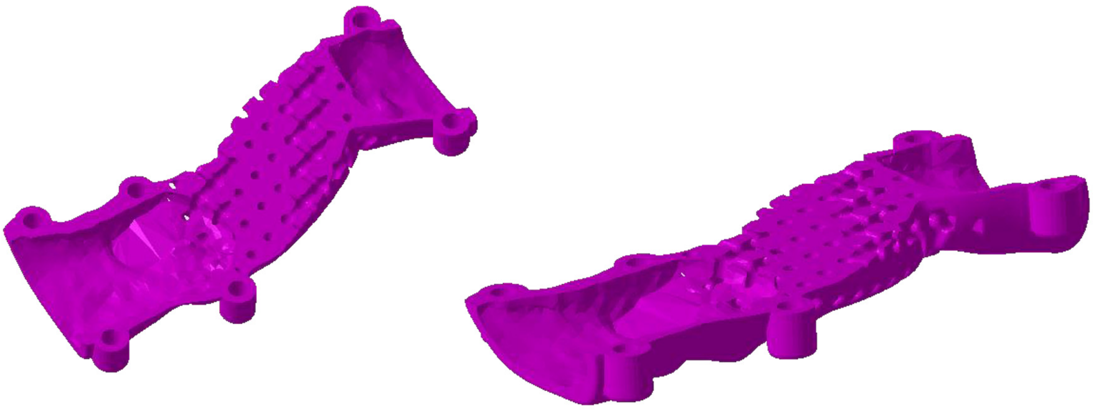

The Implant: Design Considerations

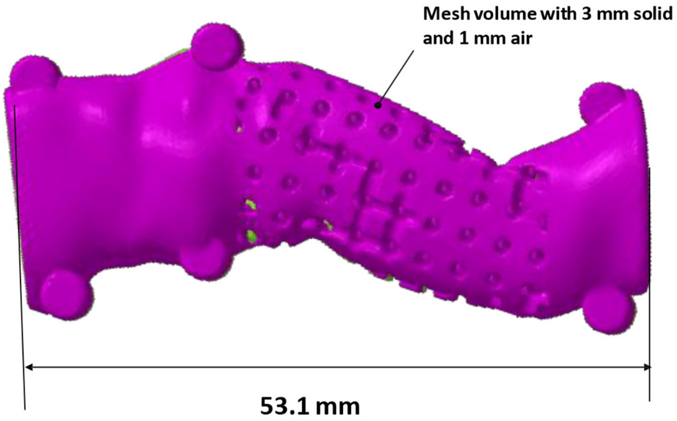

Wall thickness = 1.2mm (min) / 2.61mm (max)

The Implant:

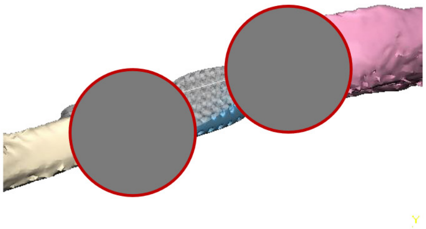

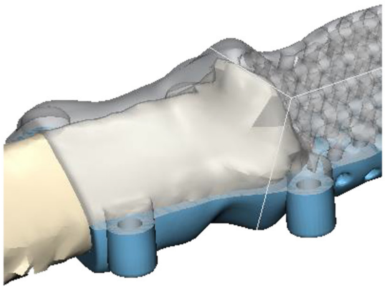

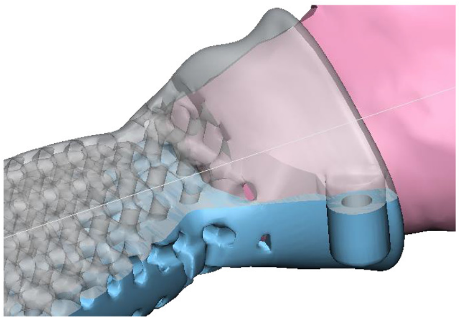

The Implant: Design Considerations

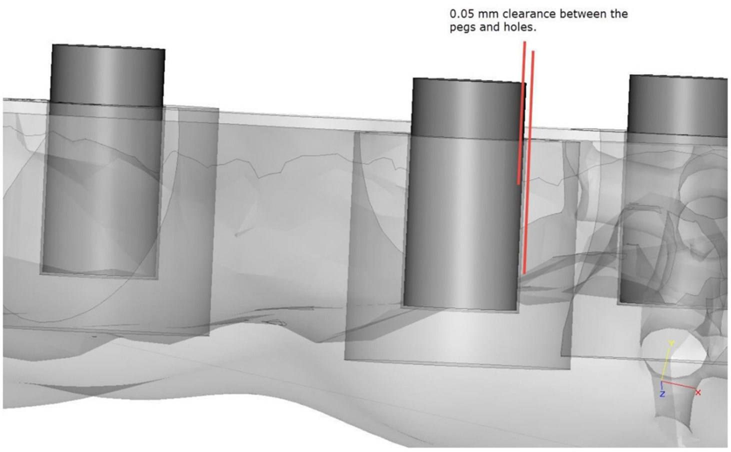

0.0.5 mm clearance pegs and holes

The Implant: Design Considerations

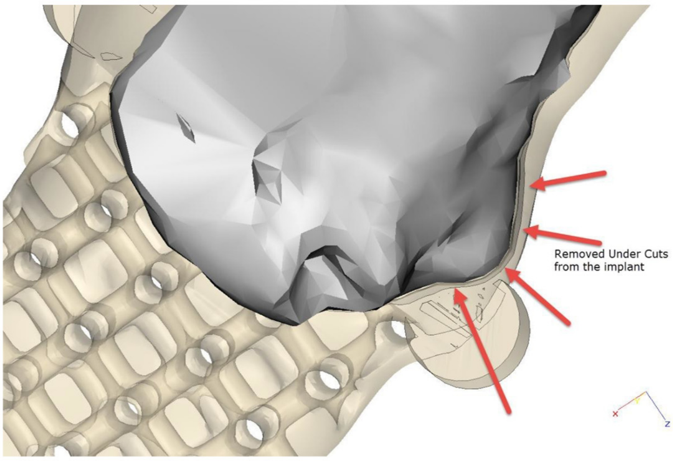

No undercuts and/or interferences are detected

The Implant: Design Considerations

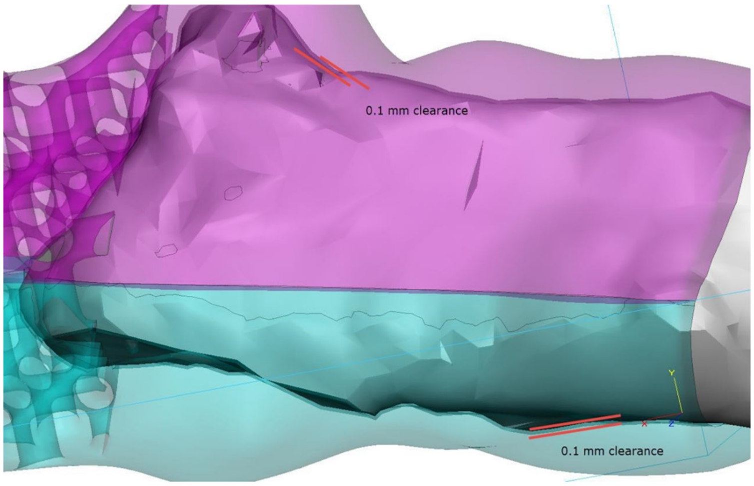

0.1 mm clearance between bone and implant

Finite Element analysis Figures S2,S3,S4

Supplement S1Figures S2a &S2b Explain Axial compression test:

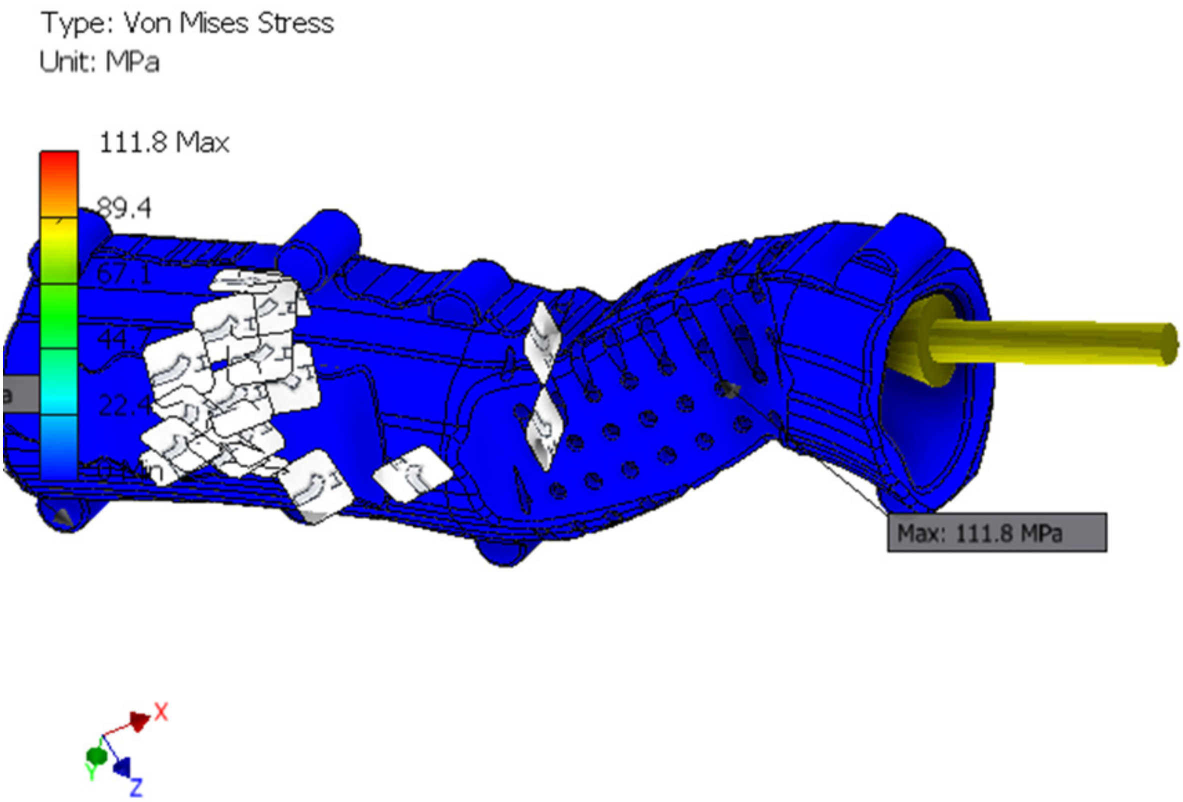

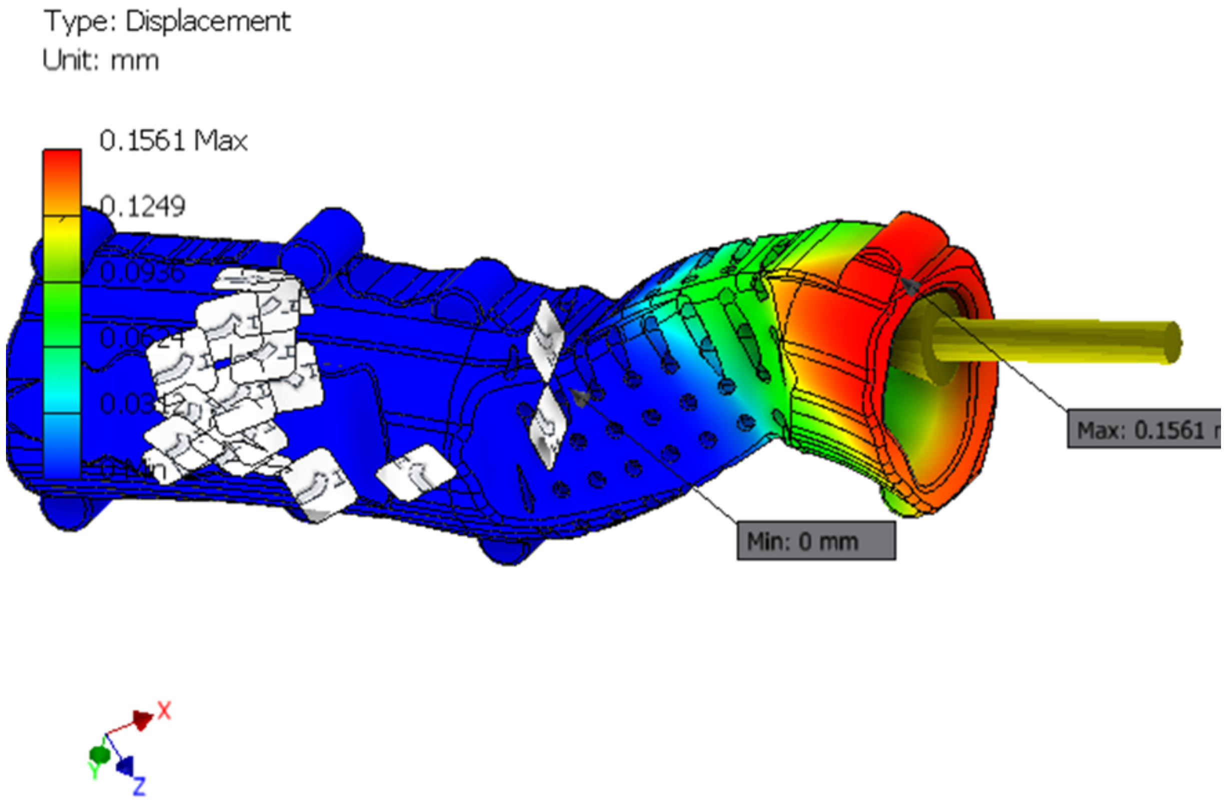

Type: Displacement
Unit: mm
0.1561 Max
0.1249
0.0936
0.0624
0.0312
Max: 0.1561
Min: 0 mm
X
Y
Z

Figures S3a &S3b Cantilever bending test:

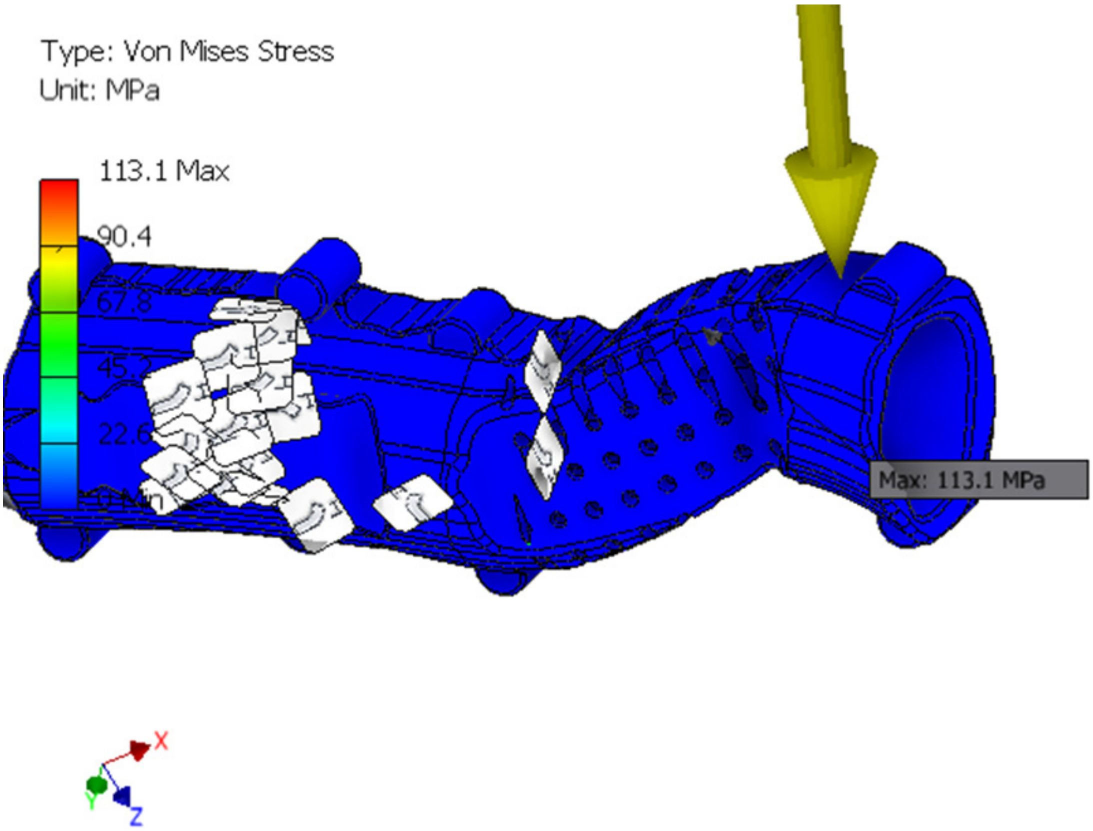

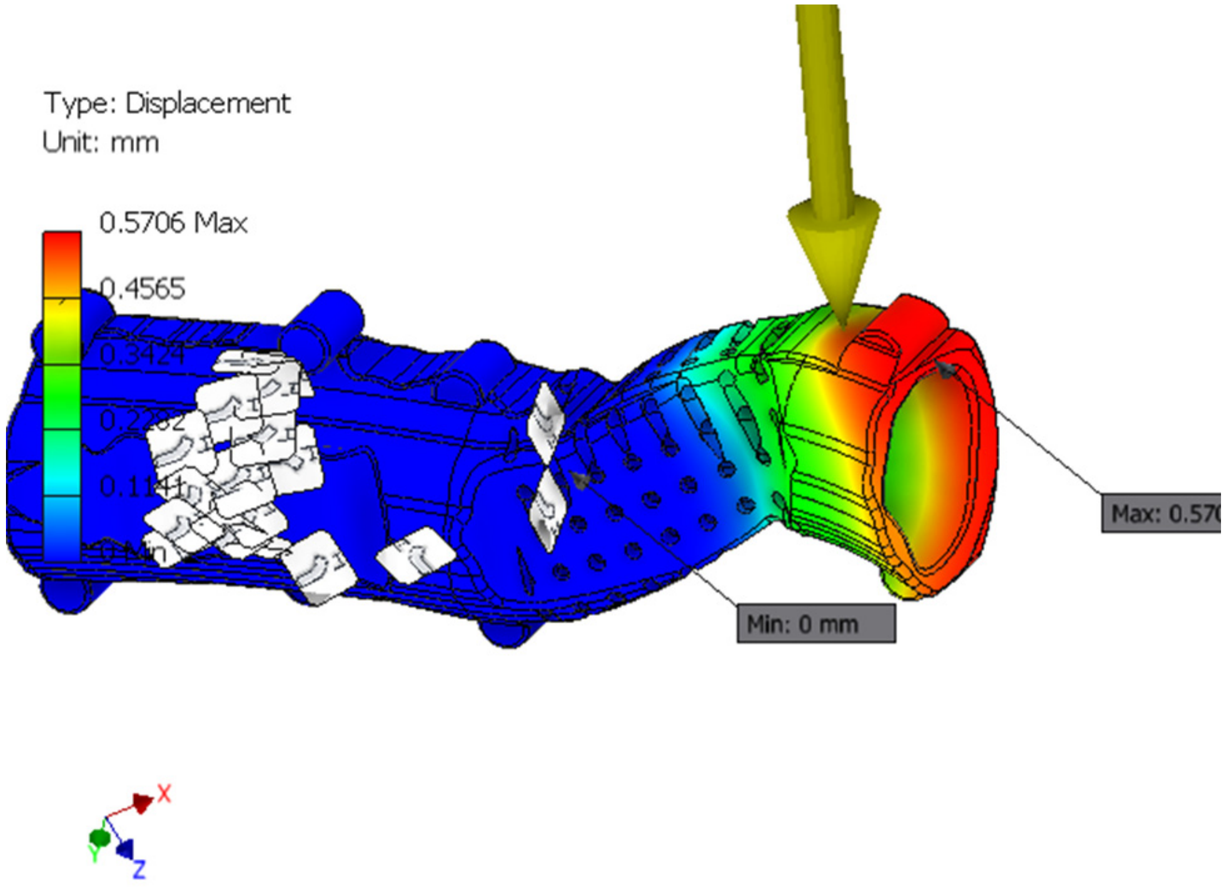

Type: Displacement
Unit: mm
0.5706 Max
0.4565
0.3424
0.2282
0.1141
Min: 0 mm
Max: 0.570
X
Y
Z

Figures S4a &S4b Explain Torsion test:

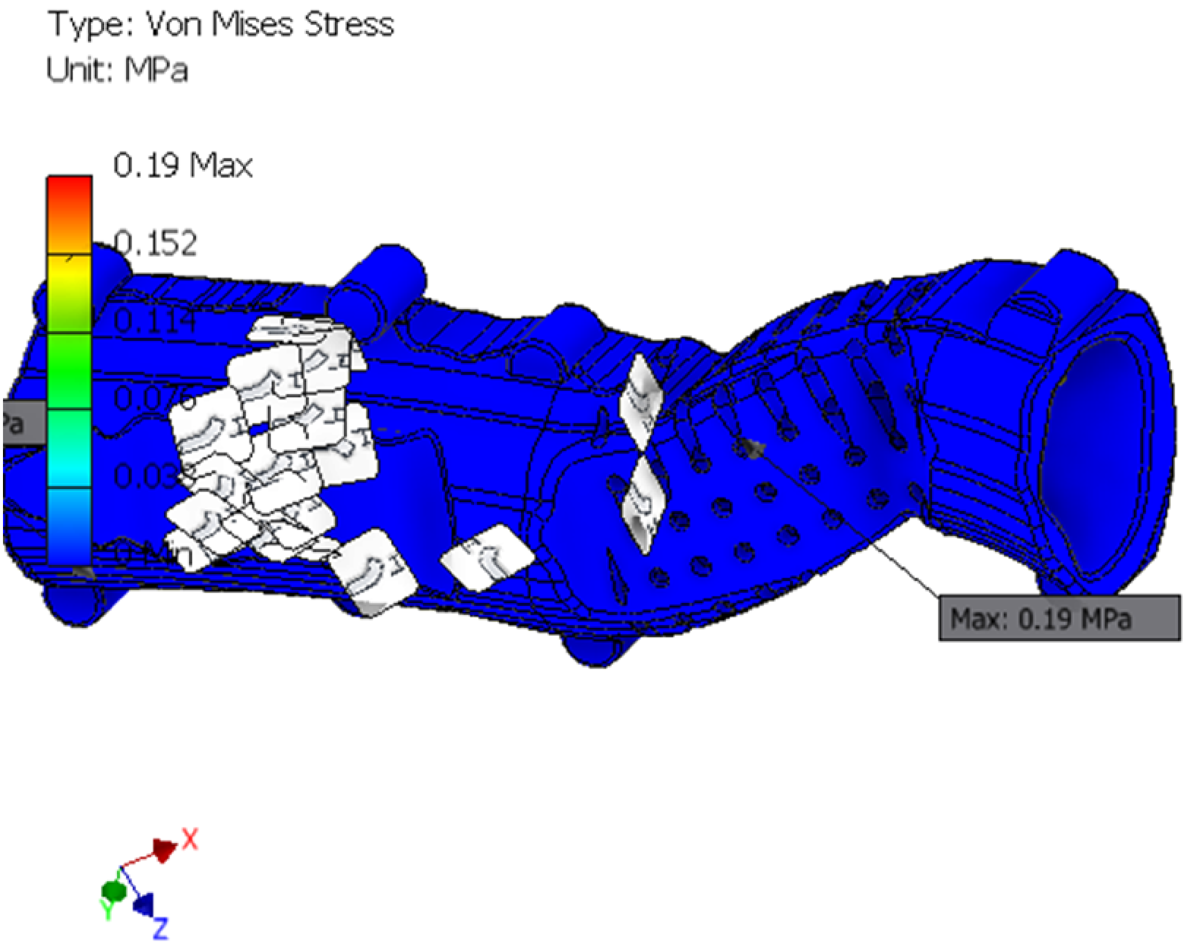

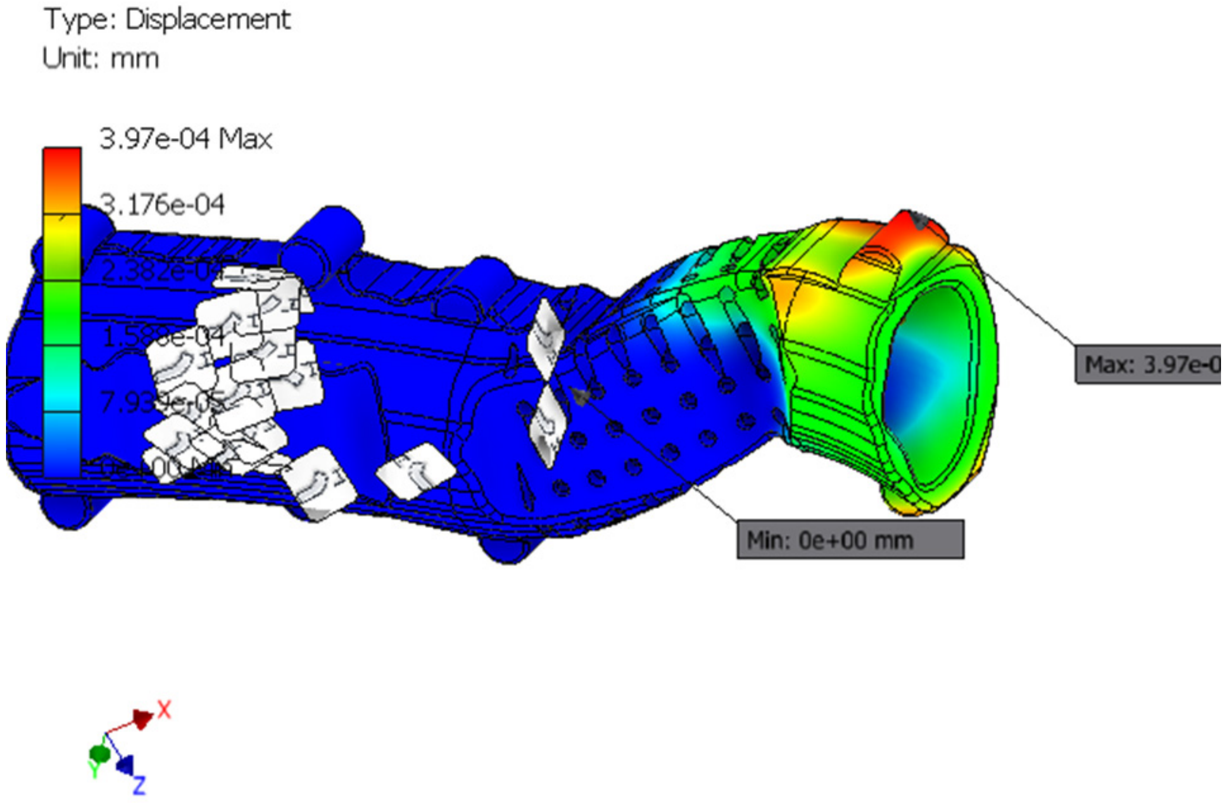

Type: Displacement
Unit: mm
3.97e-04 Max
3.176e-04
Max: 3.97e-0
Min: 0e+00 mm
X
Y
Z

Supplement S1 figure S5: Active Range of motion of the shoulder joint after 2.5 years follow-up

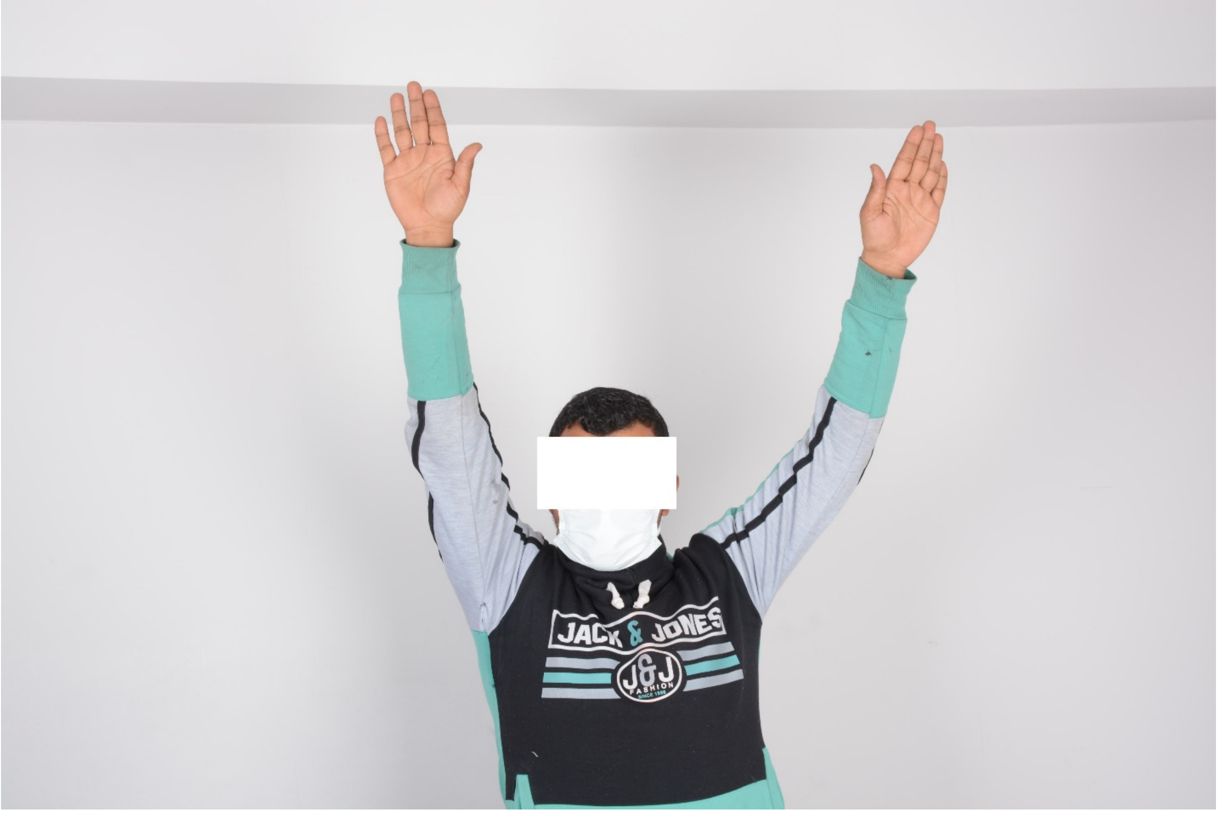

5a

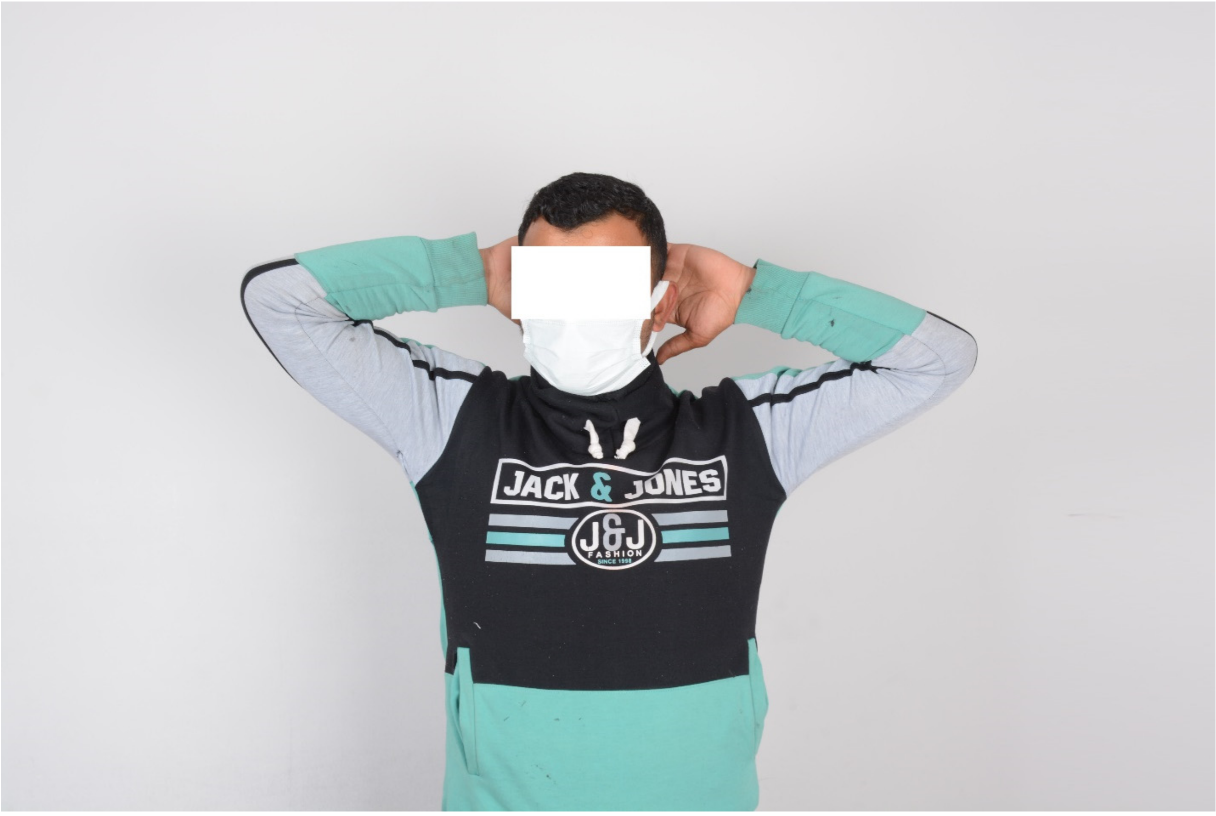

5b

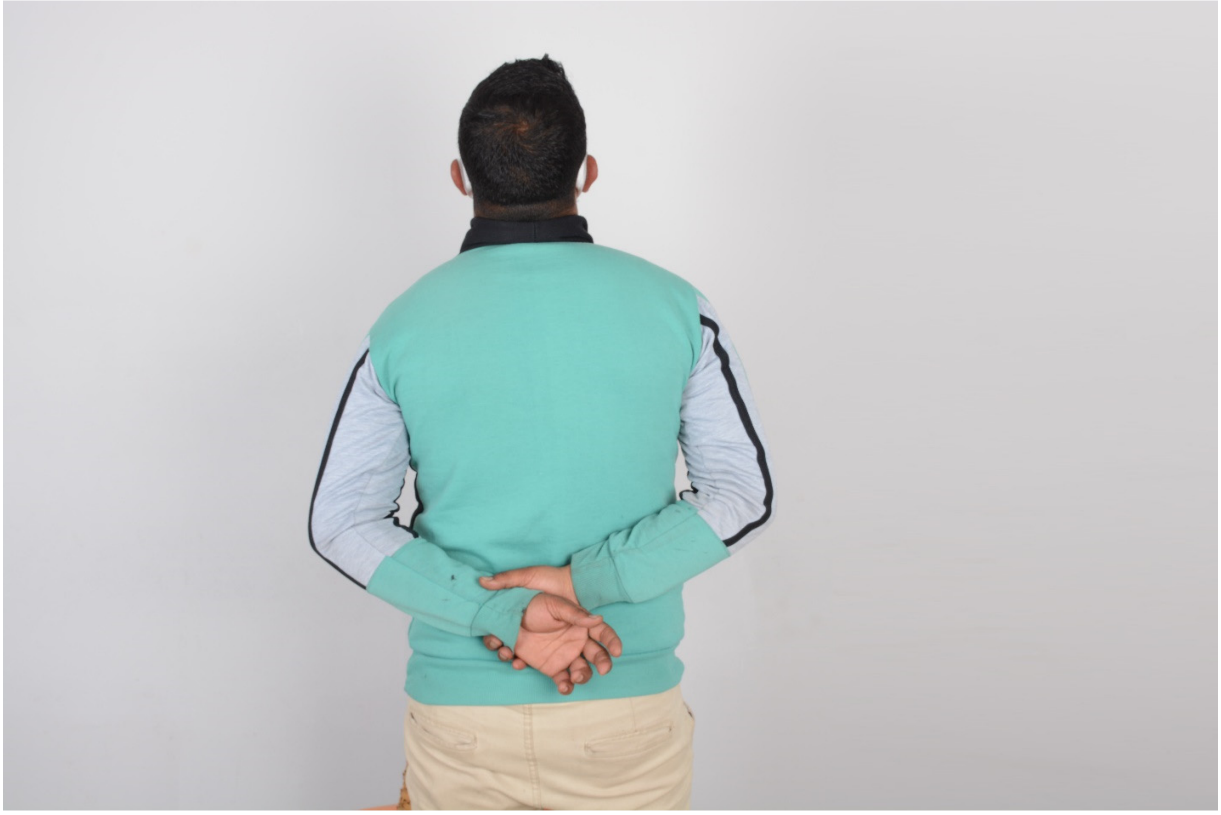

5c

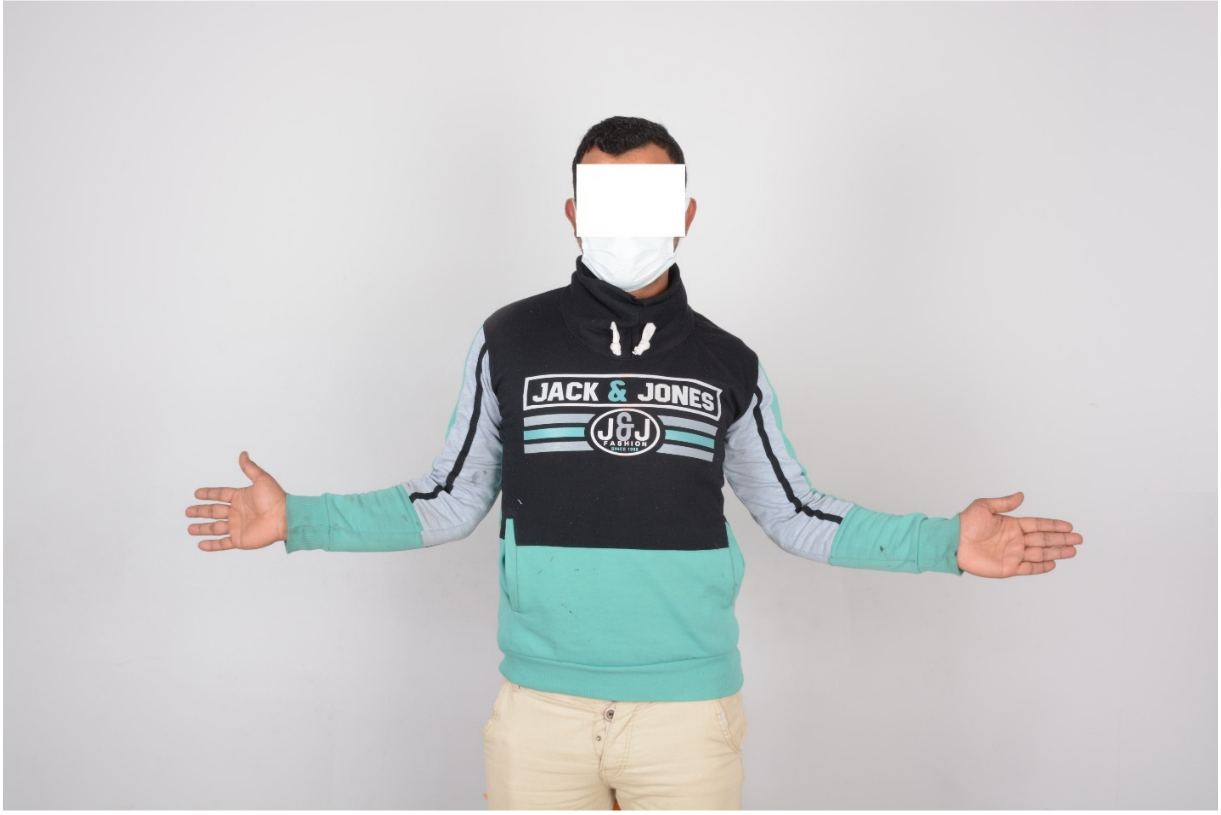

5d

Supplement S1 Figure S6 : Post operative X Ray

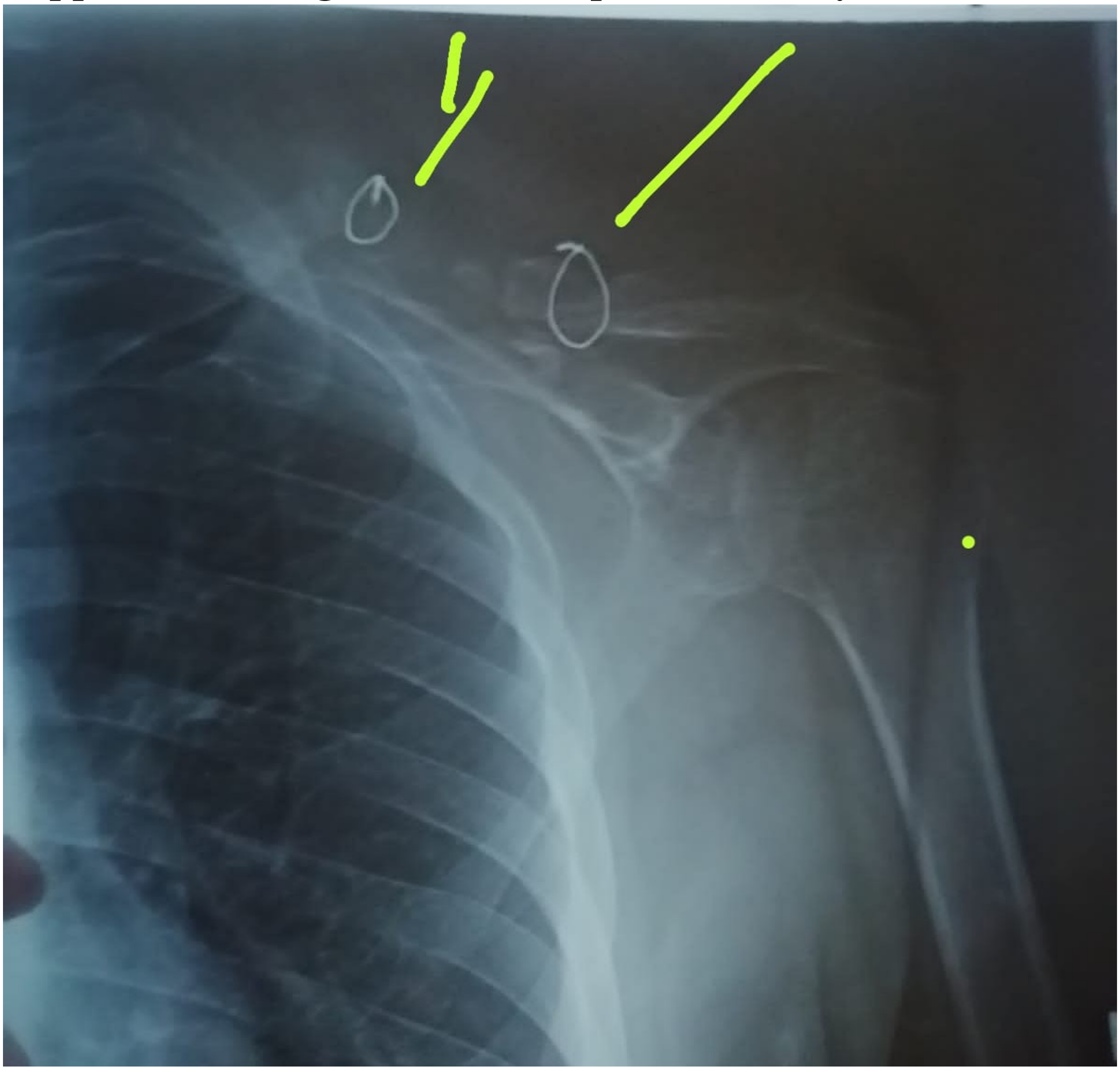

The postoperative X-ray image to demonstrate fracture healing and bone union during follow-up. We put 2 arrows on the 2 wire rings which were put to explain the site of prothesies because it does not appear at x ray, due to the polyamide material used in the patient-specific prosthesis is radiolucent and therefore does not clearly appear on standard X-ray imaging.
